# A Comparative Evaluation of the Effect of Different Endodontic Irrigating Solutions on Microhardness of Root Canal Dentin: An *in vitro* Study

**DOI:** 10.30476/dentjods.2023.98298.2071

**Published:** 2024-09-01

**Authors:** Chris Cherian Geogi, Sandeep Dubey, Palak Singh, B Rajkumar, Praveen Singh Samant, Ananya Rawat

**Affiliations:** 1 Dept. of Conservative Dentistry and Endodontics, Babu Banarasi Das College of Dental Sciences, Lucknow (U.P.), India

**Keywords:** Calcium hypochlorite, Chlorhexidine, Dentin Microhardness, Nanochitosan, Sodium hypochlorite

## Abstract

**Statement of the Problem:**

During endodontic therapy, irrigation solutions applied in the root canal may affect the physicochemical properties of the dentinal wall, thereby changing its microhardness. This may adversely affect the sealing ability and adhesion of dental materials. Therefore, many studies have focused on the search for an ideal root canal irrigant that has a minimal effect on dentinal microhardness.

**Purpose:**

This *in vitro* study was conducted to determine the changes in dentin microhardness after root canal irrigation with different endodontic irrigants.

**Materials and Method:**

Ninety-five freshly extracted maxillary central incisor teeth with straight single canals were selected. These teeth were sectioned transversely at the level of the cementoenamel junction. The working length of each tooth was determined, and canal space was prepared by the HyFlex CM rotary file system. During instrumentation, normal saline was used for irrigation. Then, teeth were split longitudinally into two segments. According to the irrigating solution employed, samples were divided into five groups (n=19): normal saline (Group A), 3% sodium hypochlorite (Group B), 2% chlorhexidine (Group C), 5% calcium hypochlorite (Group D), and 0.2% nanochitosan (Group E).3 mL of the corresponding irrigating solution was administered for total15 minutes in each prepared sample. The Vickers micro-hardness tester was then used to assess micro-hardness. The data was analyzed using one-way analysis of variance (ANOVA).

**Results:**

All tested irrigating solutions decreased the dentinal microhardness. Samples irrigated with 5% calcium hypochlorite demonstrated dentinal microhardness of 42.43±1.62, which is the lowest among all the tested groups, followed by nano chitosan, sodium hypochlorite, and chlorhexidine. Samples treated with control group (saline) demonstrated the maximum microhardness of dentin in the present study.

**Conclusion:**

Within the limitations of this research, it can be concluded that the tested novel irrigating solutions, 5% calcium hypochlorite and 0.2% nanochitosan, were more detrimental to radicular dentin microhardness when compared with conventional endodontic irrigants.

## Introduction

The eradication of microorganisms is imperative during endodontic management. To attain this, mechanical instrumentation of the infected canal space alone may not be sufficient. Therefore, irrigation of root canals by irrigating solutions is an essential part of the endodontic procedure because it facilitates the debridement and disinfection of regions inadequately cleaned by endodontic instruments [ 1 ].

During the irrigation process, these endodontic irrigants stay in close contact with the dentin surface and can influence the chemical composition of dentin and its physical characteristic features [ 2 ]. Therefore, it is important to determine the effect of the irrigating solution on radicular dentin. Changes in the calcium-phosphorus ratio have an impact on the dentin's initial ratio of organic to inorganic components, which can influence the microhardness, solubility, permeability and surface roughness of the dentin [ 2 - 3 ]. Microhardness analysis renders an arbitrary assessment of any alteration in the mineral composition of dental hard tissues. Microhardness studies are often applied to evaluate the physical characteristics of materials and to assess the hardness of teeth [ 4 - 5 ].

The irrigating solution most frequently applied during cleaning and shaping is sodium hypochlorite (NaOCl). This chemical has exquisite antimicrobial effects along with solvent capacity on vital and necrotic organic tissue [ 6 ]. Chlorhexidine (CHD) is another popular irrigation solution used during chemo mechanical debridement that has antibacterial action and no cytotoxicity [ 7 ]. However, these endodontic irrigants have little or no effect on the removal of the smear layer when applied alone [ 8 ]. This layer can be efficiently eliminated by chelating agents such as ethylene diamine tetraacetic acid (EDTA). It has been demonstrated that EDTA has certain limitations, including decreased efficacy in removing the smear layer from the apical third of canal space [ 9 ], a cytotoxic effect [ 10 ], and decreased bond strength of resin cement after its application [ 11 ]. 

These endodontic irrigants that are routinely applied in endodontic therapy have certain limitations, so the quest for an ideal endodontic irrigant continues. Nanochitosan (NCH) is a non-synthetic polycationic linear polysaccharide derived by partial deacetylation of chitin. It consists of β-(1-4)-linked d-glucosamine and Nacetyl-d-glucosamine. This polymer is non-toxic, biodegradable, biocompatible, and bioadhesive [ 12 ]. According to the literature, NCH has broad spectrum antimicrobial activity and the ability to remove smear layers [ 13 - 14 ]. However, there is limited research regarding its effect on dentin microhardness.

Calcium hypochlorite [Ca(ClO)_2_] has the potential to be used as a root canal irrigant due to its antimicrobial effects and pulp dissolution property [ 14 - 15 ]. It is relatively more stable and has a higher available chlorine ion percentage than sodium hypochlorite [ 16 ]. To the best of the author’s knowledge, there is very limited research regarding the effect of calcium hypochlorite on dentin microhardness [ 17 - 18 ].

Moreover, recent research has only focused on the effect of chelating agents on the microhardness of dentin. Conversely, in clinical conditions, chelating agents are usually applied along with other irrigants. Therefore, the aim of this study was to assess the effects of calcium hypochlorite, NCH, sodium hypochlorite, and CHD on dentin microhardness.

## Materials and Method

This research was examined and authorized by the University Ethics and Research committee in accordance with the code BBDCODS/03/2020/No.10.This research was carried out on ninety-five freshly extracted maxillary central incisor teeth with mature, intact apices and a single canal, these teeth were extracted due to periodontal reasons. Previously treated, fractured, and carious teeth were eliminated from the study. The external tooth surface was meticulously cleaned from any tissue remnants and calculus deposition, and then they were kept in 4% formalin for 72 hours.

### Specimen Preparation

By the use of a water-cooled, diamond impregnated disc, these stored teeth were subsequently sectioned transversely at the position of the cementoenamel junction. Root canal patency was verified by no. 15 K-files (Dentsply-Maillefer, Ballaigues, Switzerland) followed by a working length measurement. The length of the canal was determined by introducing a K-file in the canal until its tip was visualized at the apical foramen. The working length was then established by subtracting 1.0 mm from that length. Root canal preparation was done using the Hyflex CM nickel- titanium rotary file system (Coltene-Whaledent, Allstetten, Switzerland), to a size of 4/25, driven by an X-smart endomotor (Dentsply Maillefer, Switzerland). During instrumentation, canal space was passively delivered with 2 mL of normal saline using a side-vented 28-gauge needle, followed by the application of 5–mL of distilled water for 2 minutes as a final irrigating solution. After canal preparation, the roots were longitudinally sectioned into two halves. This was done by first preparing the grooves along the long axis of the roots with a water-cooled diamond disc (Horico, Germany), mounted on a high-speed handpiece. The roots were then cut in a buccolingual direction with a surgical chisel. 

Subsequently, these fragments were implanted in auto polymerizing acrylic resin, leaving their radicular dentin exposed. To achieve a smooth surface devoid of gradients, the dentin surface was polished using carbide abrasive papers with three gradually increasing grit sizes (400, 600, and 1200). Later, polishing was performed with an aluminum oxide paste on a rotary felt disc at a low speed [ 19 ]. 

### Group Division

Depending on the endodontic irrigants used, samples were assigned to the following groups (n= 19): Group A was normal saline (KRPL, India), Group B was 3% sodium hypochlorite (Pyrax, India), Group C was 2% CHD (PrevestDenpro, India), Group D was 5% calcium hypochlorite (Gyan Scientific Traders, India), and Group E was 0.2% NCH (Nano Wings, India). All tested irrigating solutions in their allotted group of specimens were irrigated three times continuously, 1ml for 5 minutes with a 28-gauge needle during each application. Hence, 3mL of the irrigating solution were administered in total after 15 minutes. The specimens were later washed with 20mL of distilled water to remove any remaining test solution. The specimens were blotted dry before being sent for evaluation of dentin microhardness.

### Dentin Microhardness evaluation

All samples had three indentations that ran parallel to the root canal lumen's margin. A single indentation was made at each measurement by applying a 50-g force perpendicular to the indentation surface with a dwell time of 10 seconds. The three indentations were spaced 200μm apart from one another, and the first one was produced 1000μm from the root canal entrance. The average of the outcomes for the three indentations was used to determine the hardness values for each specimen.

### Statistical Analysis

Descriptive and analytical statistics were done by using SPSS Version 24.0 (IBM Corporation, Chicago, USA). The Shapiro-Wilk test was used to evaluate the normality of the data. The data were analyzed using parametric tests because they had a normal distribution. To determine whether there were mean differences between the groups, the ANOVA test was utilized. Tukey's HSD test was used for post hoc analysis.

## Results

The group A–saline group (control) had the highest microhardness value of 58.43±2.61 followed by group C-0.2% CHD group (47.39±1.93), group B- sodium hypochlorite group (47.07±1.04) and group E-NCH group (42.62±1.76). The group D- 5% calcium hypochlorite group had the lowest microhardness value of 42.43± 1.62 (Table 1). 

**Table 1:** Mean microhardness of all the tested groups

Groups	N	Mean	S.D.	S.E.	Min.	Max.	F value	*p* value#
Normal Saline	19	58.43	2.61	0.59	53.58	62.34	230.168	<0.001†
3% Sodium Hypochlorite	19	47.07	1.04	0.23	45.38	48.90		
2% Chlorhexidine (CHD)	19	47.39	1.93	0.44	44.59	51.20		
5% Calcium Hypochlorite	19	42.43	1.62	0.37	39.90	45.31		
0.2% Nanochitosan (NCH)	19	42.62	1.76	0.40	40.11	45.80		

# *p* Value derived from one-way ANOVA test,

† significant at *p* < 0.05. N: Number of samples, S.D.: Standard Deviation, S.E.: Standard Error, CHD: Chlorhexidine, NCH: Nanochitosan

According to Table 2, when 2% CHD was compared with 3% sodium hypochlorite, a mean difference of 0.31 (95% CI: -1.37-2.00) was seen which was not significant (*p*= 0.985). Similarly, a mean difference of -0.18 (95% CI: -1.87-1.49) was observed when 5% calcium hypochlorite was compared with 0.2% NCH, which was not statistically significant (*p*= 0.998).

**Table 2:** Comparison of mean microhardness between different groups

Groups	M.D.	95% C.I.	*p* Value*	Significance
Group A v/s Group B	11.35	9.66-13.04	<0.001†	significant
Group A v/s Group C	11.03	9.35-12.72	<0.001†	Significant
Group A v/s Group D	15.99	14.30-17.67	<0.001†	Significant
Group A v/s Group E	15.80	14.11-17.48	<0.001†	Significant
Group B v/s Group C	-0.31	-2.00-1.37	0.985	Non-significant
Group B v/s Group D	4.63	2.94-6.32	<0.001†	Significant
Group B v/s Group E	4.44	2.76-6.13	<0.001†	Significant
Group C v/s Group D	4.95	3.26-6.63	<0.001†	significant
Group C v/s Group E	4.76	3.07-6.44	<0.001†	significant
Group D v/s Group E	0.18	-1.49-1.87	0.998	Non-significant

* *p* Value derived from Tukey’s HSD post hoc test,

† significant at *p* < 0.05. M.D.= mean difference. Group A- normal saline; Group B -3% sodium hypochlorite; Group C -2% chlorhexidine (CHD); Group D-5% calcium hypochlorite; Group E- 0.2% nanochitosan (NCH)

## Discussion

Microhardness assessment is one of the most commonly applied, simple, and nondestructive procedures to evaluate minute alterations in the hardness of dental hard tissues. Any alteration in the root dentin microhardness may have a negative impact on the capacity of obturating materials to seal and adhere to the dentin, which can influence the longevity of endodontically treated teeth [ 20 ]. In this investigation, microhardness assessment was done by the Vickers method, as it is less susceptible to surface conditions and provides more accurate measurements [ 21 ]. Recent studies done by Saghiri MA *et al*. [ 22 ] and Kulkarni S *et al*. [ 23 ] have employed the Vickers microhardness test to evaluate root dentin microhardness after irrigating canal space with various irrigating solutions.

According to Arul B *et al*. [ 24 ], contact time and concentration are the main determinants of how an endodontic irrigant will act. It is still unknown how long an irrigating solution should be held in root canals to eliminate the smear layer effectively. For the best possible outcome, Goldberg and Spielberg [ 25 ] suggested a longer duration of 15 minutes. In the present study, root canals were irrigated with tested endodontic irrigants for 15 minutes. Similarly, Ari H *et al*. [ 26 ] examined the effect of endodontic irrigation solutions on the dentinal microhardness by keeping the tested irrigating solution in contact with exposed dentin surfaces for 15 minutes. However, earlier research has been inconsistent with regard to the duration. While Tuncer AK *et al*. [ 27 ] assessed dentinal microhardness by keeping a contact time of 1 minute between irrigant and dentin, Akbulut MB *et al*. [ 28 ] evaluated the effects of newly proposed irrigating solutions and contemporary irrigants on the microhardness and surface roughness of human tooth surfaces by treating the dentin with an irrigating solution for 15 minutes and 30 minutes. In this investigation, to mimic the clinical conditions, root canals were irrigated with endodontic irrigating solutions by the application of syringes coupled with 28-gauge needles.

In the current study, five different irrigating solutions, 3% sodium hypochlorite, 2% CHD, 5% calcium hypochlorite, 0.2% NCH, and normal saline (control group) were evaluated for their effect on dentine microhardness. Among these irrigants, the minimum dentin microhardness in the present research was exhibited by samples irrigated by calcium hypochlorite (Table1). This result could be explained by calcium hypochlorite's capacity to improve dentin permeability, which could result in higher calcium ion sequestration and surface demineralization [ 29 ]. Demineralization refers to reduced mineral content that, in turn, influences the microhardness of the teeth. Calcium hypochlorite is available in powder form; it is formed as a root canal irrigant by mixing the powder with distilled water. According to Dutta A *et al*. [ 30 ], after ionization, an aqueous solution of calcium hypochlorite releases calcium hydroxide [Ca(OH)_2_] and HOCl. The reduced dentin microhardness caused by calcium hypochlorite can also be attributed to the effect of Ca(OH)_2_. Due to their small diameter and extremely alkaline (pH=12.5) inorganic structure, Ca(OH)_2_ molecules can infiltrate deep into the intrafibrillar structure of the mineralized collagen fibrils and alter the tropocollagen's three-dimensional conformation. As a result, the microhardness of dentin is reduced [ 31 - 32 ].

In the present study, NCH demonstrated a significant reduction in the microhardness of dentin (Table 1). The outcome of this study is in accordance with Saha SG *et al*. [ 33 ], who also reported that 0.2% NCH caused a significant reduction in the microhardness of dentin. However, the precise process by which NCH affects the dentin microhardness is not yet established. Initially, it was thought that the reduction of microhardness by NCH was due to the presence of acetic acid [ 34 ]. However, Cruz-Filho *et al*. [ 35 ] observed in their study that 5% acetic acid has minimal influence on dentin microhardness. Therefore, it can be stated that the substance, rather than the acid, may be responsible for Chitosan's action in decreasing the microhardness of dentin. Observations from the present study can be attributed to the chelating property of NCH. Due to its hydrophilic nature, NCH polymer can adhere to radicular dentin and get easily absorbed into the root canal wall [ 36 ]. Its cationic property facilitates ionic interaction between the calcium ions present in the dentinal wall and chelating chemicals. In the present study, chitosan had less effect on dentinal microhardness than calcium hypochlorite with no statistical difference between them (Table 2). This outcome can be due to covalent interaction between chitosan and the collagen in dentin, which seems to cause remineralization of demineralized dentin leading to higher dentinal microhardness [ 37 ]. 

A 3% concentration of sodium hypochlorite was chosen for this study because this is the most frequently employed concentration in clinical procedures to minimize its adverse effects [ 38 - 39 ]. In our research, the group treated with 3% sodium hypochlorite demonstrated an appropriate reduction in dentin microhardness. This result is in accordance with the findings of a recent study conducted by Elika V *et al*. [ 40 ]. Dentin consists of about 20% organic material by weight. Most of this organic material is formed by type I collagen, which considerably adds to dentin's mechanical characteristics [ 41 ]. Long peptide chains of type I collagen are broken, and their protein terminal groups are chlorinated by sodium hypochlorite to form N-chloramines, which can then be further fragmented to form new species [ 42 ]. Consequently, sodium hypochlorite irrigant can influence the mechanical properties of dentin by destruction of organic dentin components. According to Kinney *et al*. [ 43 ], the drop in dentin microhardness is due to the decrease in stiffness of the intertubular dentin matrix, which is caused by the heterogeneous distribution of the mineral content inside the collagen matrix. Haiping Xu *et al*. [ 44 ] observed that sodium hypochlorite decreases the mechanical strength of root dentin by affecting the intratubular surface close to the root canal. They also stated that as the concentration of sodium hypochlorite increases, there is a more intense effect on the mechanical properties of dentin.

The group of samples irrigated with 2% CHD solution showed the least decrease in microhardness. The outcome of the present study corroborates the finding of Aslantas EE *et al*. [ 45 ], who demonstrated that 2% CHD solution reduced the microhardness of root canal dentin. Due to its cationic nature, CHD can easily bind to anionic molecules, including the phosphates present in the hydroxyapatite lattice. Considering that the calcium carbonate complex of dentin contains phosphates, CHD can lead to alterations in the Ca/P ratio [ 46 ], which could have been the cause of the reduction in dentin microhardness in the present study.

However, there are contradictory findings regarding the effects of CHD on dentin microhardness in the literature. Dhawan R *et al*. [ 47 ] exhibited no effect of 2 % CHD on the microhardness of dentin. In contrast, Kulkarni S *et al*. [ 23 ] stated that 2% CHD as an irrigating solution was seen to have a positively strengthening impact on the microhardness of root dentin in comparison to sodium hypochlorite and EDTA, which reduced the strength of root dentin. This might be attributed to the difference in the experimental conditions, the specimen preparation methodology, and the dentin structural diversity.

## Conclusion

All of the endodontic irrigants evaluated in this study led to decrease in the root dentin's microhardness. When compared to traditional endodontic irrigants, novel irrigating solutions were more detrimental to root dentin microhardness. Further investigations are essential to assess the safety and biocompatibility of these novel irrigating solutions under clinical conditions.
